# Chemical composition, pharmacology and pharmacokinetic studies of GuHong injection in the treatment of ischemic stroke

**DOI:** 10.3389/fphar.2023.1261326

**Published:** 2023-09-07

**Authors:** Qiuyue Wang, Zhihua Yang, Liuli Guo, Zhenzhen Li, Yangxi Liu, Shaoling Feng, Yanxia Wang

**Affiliations:** ^1^ Graduate School, Tianjin University of Traditional Chinese Medicine, Tianjin, China; ^2^ Institute of Traditional Chinese Medicine, Tianjin University of Traditional Chinese Medicine, Tianjin, China; ^3^ National Clinical Research Center for Chinese Medicine Acupuncture and Moxibustion, First Teaching Hospital of Tianjin University of Traditional Chinese Medicine, Tianjin, China; ^4^ Tianjin Beichen Traditional Chinese Medicine Hospital, Tianjin, China

**Keywords:** GuHong injection, ischemic stroke, phytochemistry, pharmacology, pharmacokinetics

## Abstract

GuHong injection is composed of safflower and *N*-acetyl-L-glutamine. It is widely used in clinical for cerebrovascular diseases, such as ischemic stroke and related diseases. The objective of this review is to comprehensively summarize the most recent information related to GuHong in the treatment of stroke, including chemical composition, clinical studies, potential pharmacological mechanisms and pharmacokinetics. Additionally, it examines possible scientific gaps in current study and aims to provide a reliable reference for future GuHong studies. The systematic review reveals that the chemical composition of safflower in GuHong is more than 300 chemical components in five categories. GuHong injection is primarily used in clinical applications for acute ischemic stroke and related diseases. Pharmacological investigations have indicated that GuHong acts in the early and recovery stages of ischemic stroke by anti-inflammatory, anti-oxidative stress, anti-coagulation, neuroprotective and anti-apoptotic mechanisms simultaneously. Pharmacokinetic studies found that the main exposed substances in rat plasma after GuHong administration are hydroxysafflor yellow A and *N*-acetyl-L-glutamine, and *N*-acetyl-L-glutamine could exert its pharmacological effect across the blood-brain barrier. As a combination of Chinese herb and chemical drug, GuHong injection has great value in drug research and clinical treatment, especially for ischemic stroke disease. This article represents a comprehensive and systematic review of existing studies on GuHong injection, including chemical composition, pharmacological mechanism, and pharmacokinetics, which provides reference significance for the clinical treatment of ischemic stroke with GuHong, as well as provides guidance for further study.

## 1 Introduction

**FIGURE 1:**
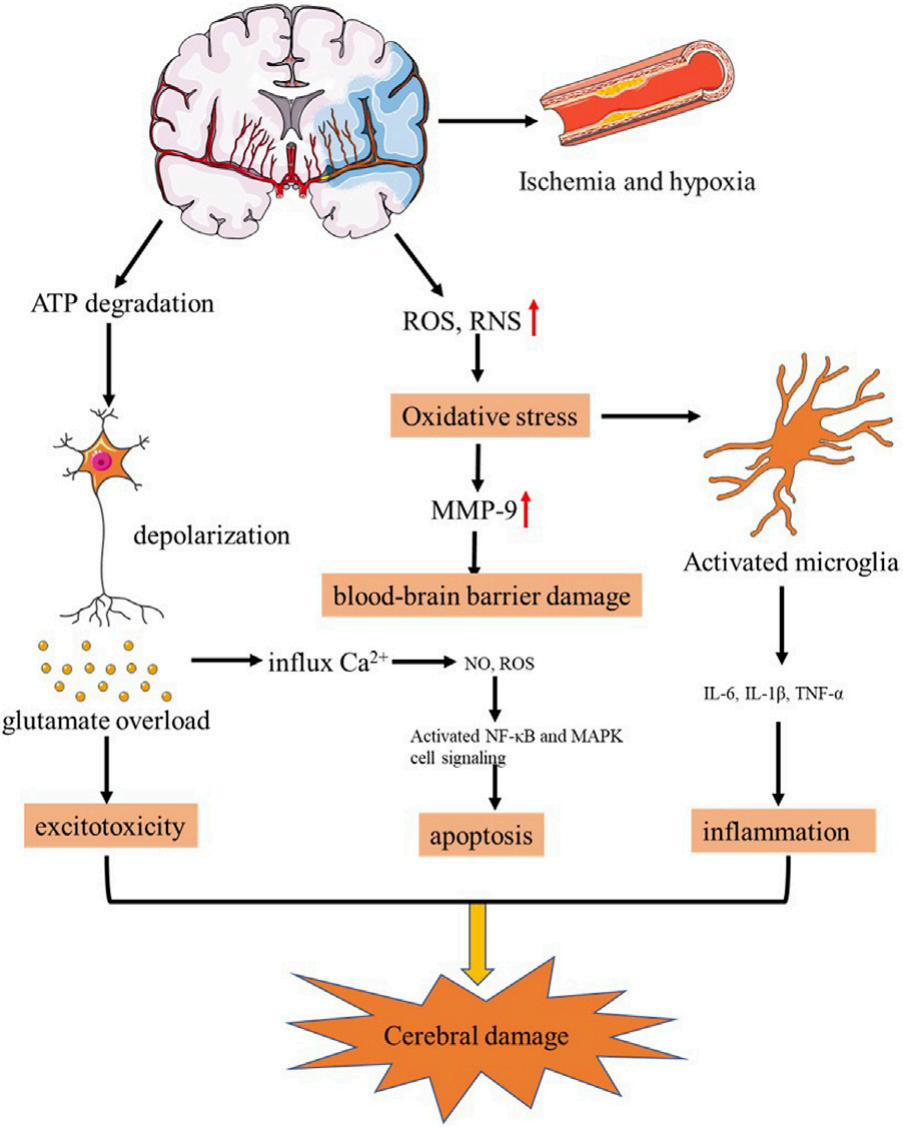
Pathogenesis of strok.

Stroke is the second highest cause of death globally and a leading cause of disability, with an increasing incidence in China. Stroke can be broadly classified into two categories: ischemic stroke and hemorrhagic stroke. Ischemic stroke is characterized by the occurrence of infarction in the brain, spinal cord, or retina, and it accounts for approximately 71% of all strokes worldwide (Campbell et al., 2019; Wu et al., 2019), which causes neuronal cell death and neurological deficits, such as learning/memory and locomotor deficiencies (Lee et al., 2018). According to TOAST (Trial of Org 10172 in Acute Stroke Treatment) criteria, ischaemic stroke was categorized into large-artery atherosclerosis, cardioembolism, small-vessel occlusion, stroke of other determined and undetermined etiology, of which arterial occlusion is responsible for the majority of strokes (Adams et al., 1993; Donnan et al., 2008). The classification of time post-stroke was based on the review of participants’ time post-stroke, and four categories regarding time post-stroke emerged: acute, subacute, post-acute, and chronic stroke (Esposito et al., 2021). The development mechanisms are complex and diverse among all possible pathological processes occurring after ischemic stroke, which includes energy failure, excitotoxicity, neuroinflammation, apoptosis and oxidative stress as shown in Figure 1 (Moskowitz et al., 2010; Campbell et al., 2019). Clinically, ischemic stroke has high mortality and disability rate. Rapid restoration of blood flow to blocked cerebral vessels is the main goal of ischemic stroke treatment and a prerequisite for neuroprotective therapy.

GuHong, a sterile, non-pyrogenic injection for intravenous administration, is prepared from *Carthamus tinctorius* flowers (Honghua in Chinese) and *N*-acetyl-L-glutamine (NAG), and approved by the Chinese National Medical Products Administration (NMPA) as an add-on therapy in the treatment of acute ischaemic stroke. Several clinical meta-analyses and clinical efficacy analyses have demonstrated the effectiveness of adding GuHong to the conventional management of acute ischemic stroke in the first week post-stroke (He et al., 2014; Liu et al., 2020). *Carthamus tinctorius* L. (C. tinctorius) or safflower, commonly called Honghua in Chinese, is an annual or biennial herbal plant in the family of Compositae. With the increasing extensiveness of studies on chemical constituents of Chinese Material Medica, investigations concerning phytochemistry have also been conducted on safflower. The dried florets of *Carthamus tinctorius* have mainly been used as injections in clinical practice. Modern pharmacological experiments have shown that *Carthamus tinctorius*, with its active compounds, possesses wide-reaching biological activities, including dilating coronary artery, improving ischemia, anti-coagulation, anti-thrombosis, anti-oxidation, and anti-inflammation, etc (Zhou et al., 2014).

Glutamine, which has traditionally been considered as a non-essential amino acid in healthy individuals, is now known to be ‘conditionally essential’ in states of serious illness or injury (Bollhalder et al., 2013; Tao et al., 2014). States of critical illness lead to significant decreases in plasma levels of glutamine and when this decrease is severe, it is correlated with increasing mortality (Oudemans van Straaten et al., 2001; Kelly and Wischmeyer, 2003; Wischmeyer, 2008). Glutamine has multiple physiological roles and functions: a precursor of nucleic acids, amino sugars, and proteins; an important nitrogen transporter; and a carrier of ammonia (Snowden et al., 2002). Glutamine acts not only as a precursor for protein synthesis and glutathione but also as a preferred fuel for the immune system and other cells involved in wound repair (Wang et al., 2010). Therefore, glutamine supplementation can protect the brain from oxidative stress during ischemic stroke (Luo et al., 2019). However, glutamine is not adequately stable in aqueous solution and is also unstable in heat treatment of liquid nutritional products (Snowden et al., 2002). NAG is a glutamine acetyl derivative which is a liquid-stable source of glutamine. As a neuropeptide, it exhibits the ability to enhance nerve cell metabolism, preserve nerve stress function, and reduce blood ammonia levels. These actions contribute to the improvement of brain function and nerve activity. (López Pedrosa et al., 2007; Zhang et al., 2015; Deng et al., 2018).

GuHong injection is composed of safflower extract and NAG. Each milliliter of the injection contains 0.5 g Carthamus tinctorius flowers (Honghua) and 30 mg NAG. GuHong injection has multi-substance and multi-target characteristics in the treatment of ischemic stroke. Pharmacological studies have shown that GuHong injection has multiple pharmacological activities, such as anti-inflammatory, antioxidant, neuroprotective, and anti-apoptotic. However, to date, there are no published comprehensive and systematic reviews on GuHong injection. In this review, studies on phytochemistry, pharmacology, pharmacokinetics and clinical application of GuHong are presented to provide comprehensive and updated information on research on GuHong in the past few decades and to investigate the therapeutic potential and safety of its components in clinical application.

## 2 Phytochemistry

GuHong injection is formulated with a combination of safflower extract and NAG. NAG is a monomer compound, whereas safflower contains a complex chemical composition. More than 300 compounds such as flavonoids, organic acids, alkaloids, and polyacetylenes have been isolated from safflower, among which flavonoid is the main active component. Given the widespread clinical usage of GuHong injection, acquiring a comprehensive understanding of its chemical composition would greatly facilitate more effective treatment of ischemic stroke and related symptoms. There are three literature references which provide the most comprehensive information on the chemical constituents present in Honghua (Zhou et al., 2014; Zhang et al., 2016; Lu et al., 2019; Xian et al., 2022). Currently, there are few studies on the composition of GuHong injection. It was found that researchers used UPLC-Q-TOF-MS/M to identify a total of 26 components in GuHong as listed in Table 1. Among these components, the majority originate from safflower, with only a small quantity of pharmaceutical excipients derived from the preparation process. It is crucial to emphasize that these excipients are generally not exhibit biological activity. Hence, apart from NAG, the main active ingredients in GuHong are predominantly from safflower.

**TABLE 1:** Chemical composition in GuHong injection.

No.	Compound	Molecular formula	Molecular weight (Da)	References
1	*N*-acetyl-L-glutamine	C_7_H_12_N_2_O_4_	188.181	Wang et al. (2022a)
2	Hydroxysafflor yellow A	C_27_H_32_O_16_	612.533	Wang et al. (2022b)
3	Quercetin-3-O-sophoroside-7-O-glucoside	C_33_H_40_O_22_	788.660	Wang et al. (2022a)
4	Notoginsenic acid beta-sophoroside	C_22_H_32_O_13_	504.184	Wang et al. (2022a)
5	Quercetin 3-glucosyl-(1->6)-glucosyl-(1->4)-rhamnoside	C_33_H_40_O_21_	772.658	Wang et al. (2022a)
6	Quercetin 3-laminaribioside	C_27_H_30_O_17_	626.148	Wang et al. (2022a)
7	Okanin 3′,4′-diglucoside	C_27_H_32_O_16_	612.533	Wang et al. (2022a)
8	Rutin	C_27_H_30_O_16_	610.518	Wang et al. (2022b)
9	Scutellarin	C_21_H_18_O_12_	462.360	Wang et al. (2022a)
10	Kaempferol-3-O-β-rutinoside	C_27_H_30_O_15_	594.518	Wang et al. (2022a)
11	Isorhamnetin 3-neohesperidoside	C_28_H_32_O_16_	624.544	Wang et al. (2022a)
12	Quercitrin	C_21_H_20_O_11_	448.377	Wang et al. (2022b)
13	Kaempferol	C_15_H_10_O_6_	286.236	Wang et al. (2022a)
14	Baicalin	C_21_H_18_O_11_	446.361	Wang et al. (2022b)
15	Safflomin C	C_30_H_30_O_14_	614.551	Wang et al. (2022a)
16	Safflower Yellow	C_43_H_42_O_22_	910.780	Wang et al. (2022a)
17	Syringin	C_17_H_24_O_9_	372.367	Wang et al. (2022a)
18	Meglumin	C_7_H_17_NO_5_	195.214	Wang et al. (2022a)
19	Guanosine	C_10_H_13_N_5_O_5_	283.241	Wang et al. (2022a)
20	L-phenylalanine	C_9_H_11_NO_2_	165.189	Wang et al. (2022a)
21	p-Hydroxy benzaldehyde	C_7_H_6_O_2_	122.032	Wang et al. (2022b)
22	Neochlorogenic acid	C_16_H_18_O_9_	354.309	Wang et al. (2022a)
23	4-O-beta-D-glucosyl-4-coumaric acid	C_15_H_18_O_8_	326.299	Wang et al. (2022a)
24	Chlorogenic acid	C_16_H_18_O_9_	354.309	Wang et al. (2022b)
25	Cryptochlorogenic acid	C_16_H_18_O_9_	354.309	Wang et al. (2022a)
26	Gallic acid	C_7_H_6_O_5_	170.120	Wang et al. (2022b)

### 2.1 Flavonoids

**FIGURE 2:**
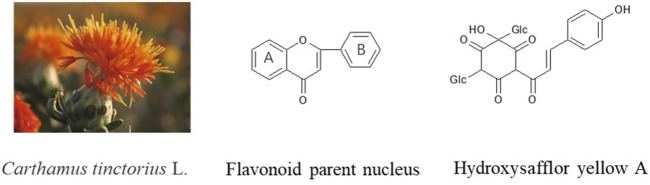
Chemical structure of flavonoids from Safflower.

The flavonoids found in safflower are an important class of pharmacologically active ingredients and the most reported class of major compounds, which can be classified as quinonechalcones, flavonols, flavonoids and dihydroflavonoids according to their chemical structure. Its structure is distinguished by the oxidation of the A ring of the parent nucleus of the flavonoid (Figure 2), in the form of quinone or quinone analogues, and all of them are C- glycosides (Yue et al., 2013). The specific ingredients are as follow: hydroxysafflor yellow A, hydroxysafflor yellow B, hydroxysafflor yellow C (Feng et al., 2013; Yue et al., 2014), safflor yellow A, safflor yellow B (Zhou et al., 2014), saffloquinoside A, saffloquinoside B, saffloquinoside C, saffloquinoside D, saffloquinoside E (Jiang et al., 2013; Yue et al., 2014), cartormin, isocartormin (Li F. et al., 2017), safflomin A, safflomin C (Zhao et al., 2022), etc. The most representative of flavonoids is hydroxysafflor yellow A, which is considered as a quality marker in the Pharmacopoeia of the People’s Republic of China (2020). In addition, another major class of flavonoid components in safflower is flavonols with quercetin and kaempferol as the parent structure (Jin et al., 2008; Xie et al., 2016), mostly in the form of O-glucosides. They are usually substituted by monosaccharide (mainly including glucose, rhamnose, glucuronide) or disaccharide (mainly including rutinose, sophorose) at the 3, 6, and 7 positions of their original nucleus (Jin et al., 2008), such as quercetin-3-*O*-β-D-glucoside, quercetin-7-*O*-β-D-glucoside, kaempferol-3-*O*-β-D-glucoside, kaempferol-3-*O*-β-rutinoside, kaempferol-3-*O*-β-sophoroside, 6-hydroxykaempferol, 6-hydroxykaempferol-3-*O*-β-D-glucoside, 6-hydroxykaempferol-7-*O*-β-D-glucoside (Hattori et al., 1992; Kazuma et al., 2000; Hu et al., 2013; Lu et al., 2019). Apart from the above compounds, there are flavonoids and dihydroflavonoids in safflower.

### 2.2 Organic acids

As a class of acidic organic compounds containing carboxyl groups, organic acids are also one of the pharmacologically active components in safflower. Currently, the organic acids isolated from safflower mainly include *p*-coumaric acid, coumaric acid glycosides (Zhou et al., 2008), chlorogenic acid, butyric acid, *p*-hydroxybenzoic acid, ferulic acid, gallic acid and caffeic acid (Jiang et al., 2008b; Lu et al., 2019).

### 2.3 Alkaloids

The alkaloids extracted and isolated from safflower contain indole rings and *p*-hydroxycinnamamide groups in their molecules, which are 5-hydroxytryptamine derivatives of indole alkaloids and have anti-oxidant pharmacological activities. Such alkaloids include *N*-feruloyl-5-hydroxytryptamine,*N*-(p-coumaroyl)-5-hydroxytryptamine, *N*-feruloyltryptamine, 4,4″-bis(*N*-p-coumaroyl)5-hydroxytryptamine, 4,4″-bis(*N*-p-coumaroyl)-5-hydroxytryptamine (Jiang et al., 2008b; Zhou et al., 2014).

### 2.4 Polyacetylenes

The polyacetylene compounds in safflower are mainly ten-, thirteen- and fourteen-carbon compounds with unsaturated double bonds mainly in trans configuration (Li et al., 2021). These components are unstable, prone to decomposition and conformational transformation when exposed to light, and have relatively weak anti-inflammatory effects (Xian et al., 2022), such as (2E, 8Z)-decadiene-4,6-diyne-1-ol-1-*O*-β-D-glucopyranoside, (2E,8E, 10E)-tridecatriene-4,6-diyne-1,12,13-triol-1-*O*-β-D-glucopyranoside, (2E)-tetradecaene-4,6-diyne-1,10,14-triol-1-*O*-β-D-glucopyranoside, (2E, 8E)-tetradecadiene-4,6-diyne-1,12,14-triol-1-*O*-β-D-Glucopyranoside, (2Z, 8Z)-tetradecadiene-4,6-diyne-1,12,14-triol-1-*O*-β-D-glucopyranoside, (2Z, 8E)-tetradecadiene-4,6-diyne-1,12,14-triol-1-O-β-D-Glucopyranoside, (2E, 8Z)-tetradecadiene-4,6-diyne-1,12,14-triol-1-*O*-β-D-glucopyranoside (Liu et al., 2017), etc.

### 2.5 Spermidines

Spermidines are low molecular weight aliphatic carbons containing three amine groups (Li et al., 2013), and the main spermidine compounds isolated from safflower are safflospermidine A, safflospermidine B, N^1^,N^5^,N^10^-(Z)-tri-p-coumaroyl spermidine, N^1^,N^5^,N^10^-(E)-tri-p-coumaroyl spermidine, N^1^,N^5^-(Z)-N^10^-(E)-tri-p-coumaroyl spermidine (Jiang et al., 2008a; Zhao et al., 2009; Li et al., 2016).

## 3 Pharmacological mechanisms of GuHong injection for ischemic stroke treatment

Pharmacological investigations have observed that GuHong exhibits brain protective effects on ischemic by reducing thrombosis, anti-oxidation, inhibiting inflammation and apoptosis, maintaining mitochondrial integrity, and improving microvasculature and microcirculation. GuHong is comprised of safflower and NAG, which is a glutamine acetyl derivative and a stable form of glutamine. When GuHong is introduced into the body, NAG is mainly metabolized into glutamine to play its drug effect. This section focuses on a review of pharmacological mechanisms of GuHong, safflower and its chemical composition, NAG and its metabolites, which are shown in Table 2.

**TABLE 2:** Pharmacological effects of GuHong injection.

Pharmacological activity	Effective dose	Animal/Cell	Route	Positive control	Effects	Mechanisms	Application	References
Anti-inflammatory	2.5, 5, 10 mL/kg	Male SD rats MCAO model	i.p	Nimodipine 10 mL/kg	Inhibition inflammatory response to ameliorative effect on cerebral I/R injury in rats	NO ↓	*In vivo*	Ai et al. (2017)
						iNOS ↓		
						TNF-α ↓		
						IL-1β ↓		
						MPO ↓		
						CPR ↓		
						ICAM-1 ↓		
						NF-κB p65 ↓		
	2.5, 5, 10 mL/kg	Male C57BL/6 J mice MCAO model	i.p	Minocycline 45 mg/kg	Decreased the abnormally elevated concentrations of proinflammatory cytokines in damaged cortex tissues	TNF-α ↓	*In vivo*	Wang et al. (2022a)
						IL-1β ↓		
						IL-6 ↓		
	1.25, 5 mL/kg	Male SD rats MCAO model	i.p	Ginaton 8 mL/kg	Decreased these inflammatory cytokines enhance the expression of molecules that maintain the blood-brain barrier	C5AR1 ↓	*In vivo*	Zhang et al. (2022a)
						C5A ↓		
						CASP3 ↓		
						8-OHdG ↓		
						TNF ↓		
						IL-1β ↓		
						IL-6 ↓		
						ICAM-1 ↓		
						MMP-9 ↓		
						MCP-1 ↓		
						TIMP1 ↑		
						JAM-A ↑ laminin ↑		
Anti-oxidative stress	2.5 mL/kg	Male SD rats MCAO model	i.p	Ginaton 8 mL/kg	enhanced anti-oxidant systems prevented ASK1 activation and suppressed subsequent p38 and JNK cascade-mediated apoptosis	GSH ↑	*In vivo*	Zhang et al. (2020)
						Trx ↑		
						Nrf2 ↑		
	5 mL/kg	Male SD rats cerebral ischemia/reperfusion	i.v./i.g. (postive)	Nimodipine (i.g)9.375 mg/kg	Increase the anti-oxidant stress and decrease the apoptotic rate	SOD ↑	*In vivo* and *in vitro*	Wang et al. (2021)
						MDA ↓		
	2.5, 5, 10 mL/kg	Male SD rats MCAO model	i.p	N.d	Enhance anti-oxidant factors and related enzymes to prevent cell damage	SOD ↑	*In vivo*	Zhou et al. (2021)
						MDA ↓		
						LDH ↓		
						MMP-9 ↓		
Anti-coagulant and anti-thrombotic	2.5, 5, 10 mL/kg	Male SD rats MCAO model	i.p	Nimodipine 10 mL/kg	Prevent thrombosis and regulate local blood flow	t-PA ↑	*In vivo*	Shu et al. (2014)
						6-keto-PGF_1α_ ↑		
						PAI ↓		
						TXB_2_ ↓		
Neuroprotection	2.5, 5, 10 mL/kg	Male SD rats MCAO model	i.p	N.d	Repair brain microvascular and mitochondria, maintain the normal function of nerve cells	BFGF ↑	*In vivo*	Zhou et al. (2021)
						VEGF ↑		
						TGF-β1 ↑		
Anti-apoptosis	2.5, 5, 10 mL/kg	Male C57BL/6 J mice or Male SD rats MCAO model	i.p	Minocycline 45 mg/kg	Regulate P13K/AKT pathways to maintain anti-apoptotic, cerebral microvascular and mitochondrial integrity	Cyt-c ↓	*In vivo*	Zhou et al. (2021); Wang et al. (2022a)
						Bax ↓ caspase-3 ↓		
						Bc-2 ↑		
	10 mL/kg	Male SD rats MCAO model	i.p	Nimodipine 10 mL/kg	Through PKC/HIF-1α pathway to ameliorate cerebral I/R injury	HIF-1α↓	*In vivo*	Yu et al. (2021)
						PKC ↓		
						Erythropoietin ↓		

Note: MCAO, middle cerebral artery occlusion.

**FIGURE 3:**
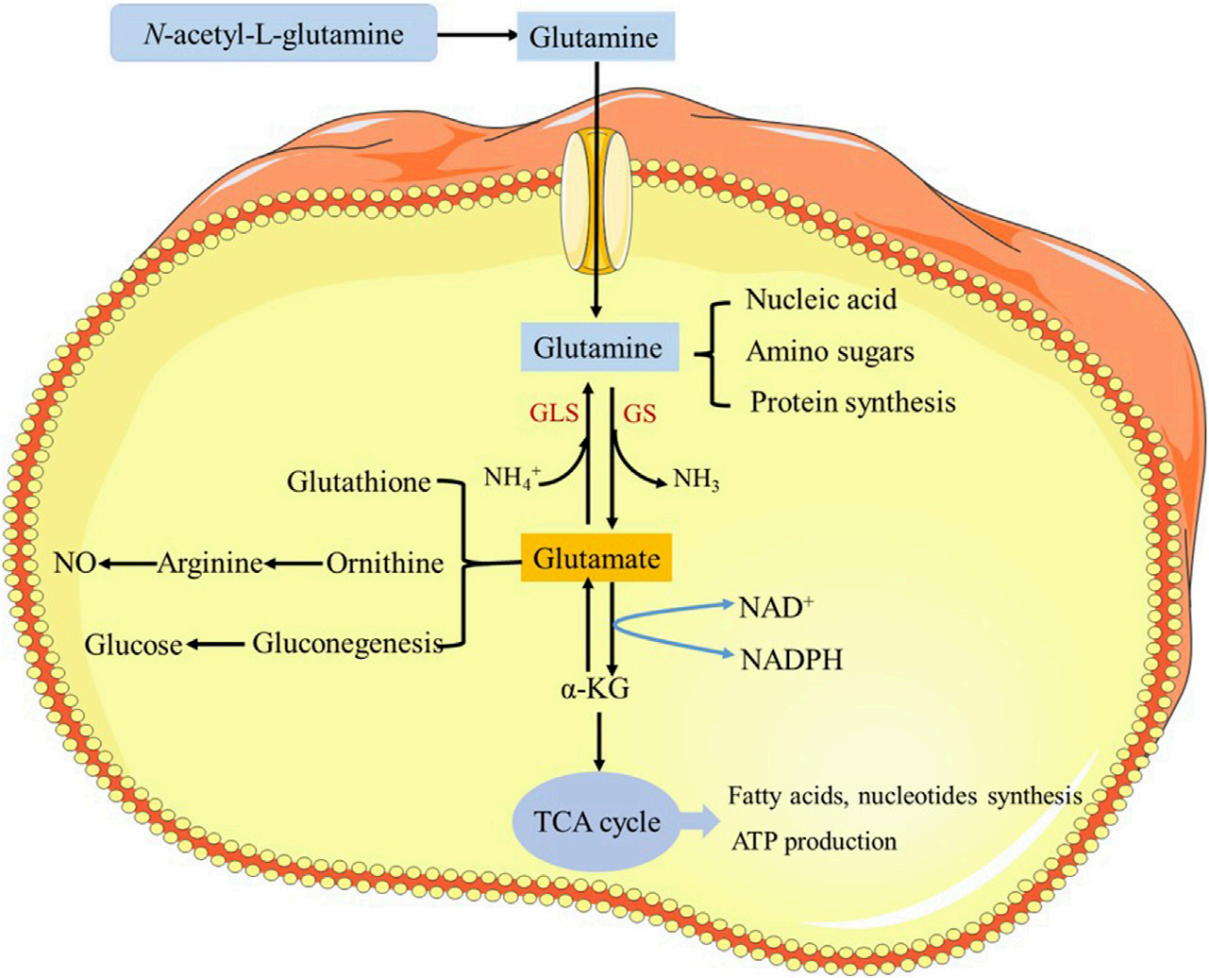
Pharmacological mechanism of *N*-acetyl-L-glutamine.

Safflower is one of the commonly used drugs in the treatment of ischemic cardiovascular and cerebrovascular diseases, which has the effect on activating blood and removing stasis (Yu et al., 2019). It is mainly used in traditional medicine for amenorrhea, dysmenorrhea and lochia. Recent studies have showed that flavonoids are the main bioactive components in safflower, which have anti-inflammatory, anti-oxidant, anti-apoptosis, anti-cerebral ischemic reperfusion injury and protection of cardiovascular and cerebrovascular effects (Asgarpanah and Kazemivash, 2013). However, there are relatively few reports on the pharmacodynamics of NAG. Since NAG is a derivative of glutamine, it acts primarily as glutamine after introducing into the body. Clinically, glutamine plays a versatile role in cellular metabolism. It acts as a crucial nitrogen source for cells, serving as an essential precursor for the synthesis of proteins and nucleic acids. Additionally, it enhances immunity by promoting replication of immune cells and maintaining their functionality. Glutamine also aids in reducing muscle catabolism and improving nitrogen balance. (Andrews and Griffiths, 2002; Cruzat et al., 2018). Furthermore, it exhibits antioxidant effects by promoting the synthesis of glutathione, reducing oxygen free radicals, and alleviating inflammatory responses. (Amores Sanchez and Medina, 1999); Lastly, glutamine serves as a cellular energy source by acting as a substrate for the tricarboxylic acid (TCA) cycle, (Xiao et al., 2016), generating adenosine triphosphate (ATP) to support cellular functions and safeguard intercellular material metabolism. (Yoo et al., 2020). Its pharmacological mechanisms are shown in Figure 3.

### 3.1 Anti-inflammatory

After ischemic stroke occurs, the accumulation of reactive oxygen species (ROS), inflammatory factors, and necrotic cells will trigger an inflammatory response (Khoshnam et al., 2017). The main pathological mechanism of ischaemic stroke is the excessive release of inflammatory factors. Many studies have shown that post-stroke neuroinflammation is an important factor affecting long-term ischemic prognosis. Thus, neuroinflammation has been regarded as a vital pathological process following cerebral ischemia-reperfusion injury (Yu et al., 2022).

Research finding indicate that the administration of GuHong can significantly decrease the levels of nitric oxide (NO), inducible NO synthase (iNOS), myeloperoxidase (MPO), interleukin-1β (IL-1β), TNF-α (tumor necrosis factor-α) and C reactive protein (CRP) in serum induced by ischemia reperfusion injury in rats. Further, histological examination by H&E staining revealed that after the intervention of GuHong in rats, the cell outlines appeared distinct and a substantial number of neurons were survived. The immunohistochemical staining revealed that GuHong administration significantly attenuated expressions of intercellular cell adhesion molecule-1 (ICAM-1) and nuclear factor-κB p65 (NF-κB p65) in rat brain tissues to exert anti-inflammatory effects (Ai et al., 2017). Besides, the interaction between the active components of GuHong and NF-κB p65 was determined by molecular docking. GuHong and its active substances partially prevent thrombosis and ischemic stroke by regulating NF-κB mediated inflammatory responses (Wang et al., 2022b). According to the results of immunofluorescence and ELISA, the administration of GuHong (10 mL/kg) to mice with ischemic stroke decreased NF-κB p65 nuclear translocation and regulated the content of pro-inflammatory factors including TNF-α, IL-6, and IL-1β in the damaged cortical tissue of mice with subacute stroke (Wang et al., 2022a). Moreover, C5ar1 (CD88) is considered to be an important potential therapeutic target for the regulation of inflammation in ischemic stroke. Experiments demonstrated that the administration of GuHong could lead to a decrease of C5AR1, C5A, CASP3, 8-OHdG, as well as inflammatory factors covering IL-1β, TNF, IL6, ICAM-1, MMP9, MCP-1 in MCAO rat model. Additionally, GuHong also enhanced the expression of tissue inhibitor of metalloproteinases 1 (TIMP1), junctional adhesion molecule 1 (JAM-A) and laminin to regulated cell growth and differentiation (Zhang J. J. et al., 2022).

The content of safflower in GuHong injection is equivalent to 0.5 g raw drug, in which hydroxylsafflower yellow A (HSYA) is used as the quality control component (0.410–0.437 mg/mL). Experimental data have demonstrated that safflower extract also has anti-inflammatory effects on brain infarct areas by reducing free radical levels in the blood and inhibiting the expression of TNF-α and IL-1β (Fu et al., 2016). Different extraction methods of safflower can affect the type and content of chemical components, resulting in different drug effects. Safflower aqueous and methanol extracts were shown to inhibit inducible iNOS and cyclooxygenase-2 (COX-2) protein expression and to diminish LPS-induced release of NO, prostaglandin E2 (PGE2) and IL-1β as well as to translocated nuclear factor (red-like derivative 2)-like 2 (Nrf2) from the cytoplasm to the nucleus and significantly reduced NF-κB binding and NF-κB luciferase activity. In addition, safflower methanol extract also significantly attenuated tumor necrosis factor (TNF-α)-mediated vascular cell adhesion protein 1 (VCAM-1) expression in endothelial cells, thus, regardless of the extraction method, safflower exhibited significant anti-inflammatory effects (Jun et al., 2011; Wang et al., 2011). HSYA is the main representative component of safflower in GuHong injection. In the literature, HSYA has been reported to decrease brain infarct volume in ischemia-reperfused rats, increase GSK-3β phosphorylation levels, downregulate the expression of several key pro-inflammatory cytokines, and inhibit the activation of iNOS, NF-κB and caspase-3, These actions collectively contribute to its anti-inflammatory and anti-apoptotic effects (Ye and Gao, 2008; Yang et al., 2020). In addition, HSYA inhibits LPS-induced reduction in pro-inflammatory factor levels and suppress VSMC proliferation and migration by inhibiting the TLR-/Rac1/Akt pathway (Yang et al., 2015). Under the condition of pathological injury, oral supplementation with glutamine can significantly reduce TNF-α and IL-1β (Cruzat et al., 2014).

### 3.2 Anti-oxidative stress

Due to high oxygen demand and limited anti-oxidant capacity, the brain is quite sensitive to hypoxia and susceptible to oxidative damage. Ischemia-reperfusion leads to the production of highly harmful ROS, and then trigger oxidative stress (OS), which is responsible for most of the ischemia-reperfusion-induced brain damage. In addition, OS can lead to apoptosis, autophagy, and necrosis of brain cells (Rodrigo et al., 2013; Orellana Urzúa et al., 2020).

The triple anti-oxidant system of Nrf2, glutathione (GSH) and thioredoxin (Trx) could be enhanced by GuHong, which were more effective than its two combinations in ameliorating oxidative damage after brain I/R. Moreover, GuHong enhanced the triple anti-oxidant system while blocked the activation of ASK1 and subsequently inhibited the activation of p38 and JNK signaling cascades, preventing oxidative damage and apoptosis (Zhang et al., 2020). Glutathione-s-transferase (GST) is a detoxification enzyme that exerts cellular detoxification by catalyzing the reaction of reduced glutathione (GSH) with electrophilic reagents substance (Ji et al., 2019). GuHong can increase the expression levels of GST P mRNA and protein, and regulate oxidative stress (Chen et al., 2023). GuHong injection increases SOD (superoxide dismutase) levels and reduces MDA (malondialdehyde) levels in patients with ischemia-reperfusion to prevent oxidative damage (Wang et al., 2021; Zhou et al., 2021).

Under pathological conditions, brain tissue experiences ischemia and hypoxia, leading to the excessive production of reactive oxygen species and an accumulation of free radicals. This process results in brain lipid peroxidation and exacerbates injury to the brain tissue. Flavonoids are the main components of safflower extracts in GuHong and are active in scavenging radicals including O^2-^, –OH, and DPPH in a dose-dependent manner (Han et al., 2010). HSYA, as one of the main quinonechalcones in GuHong, can inhibit Ca^2+^ and H_2_O_2_ induced swelling of rat brain mitochondria and decreased the production of ROS. HSYA treatment can significantly reduce the MDA content in the ipsilateral hemisphere and serum, and increase the activity of SOD and total anti-oxidant capacity (Wei et al., 2005; Tian et al., 2008). The tripeptide GSH is the most important intracellular anti-oxidant. Glutamine is an important precursor compound for glutamate, and the synthesis of GSH depends on the supply of glutamine to glutamate. Glutamine, as a precursor for glutathione synthesis, is clinically added to patients against oxidative stress (Amores Sanchez and Medina, 1999). NAG can also enhance the anti-oxidant system and effectively improve the oxidative damage of ischemia reperfusion, although less effective than combined safflower extract (Zhang et al., 2020).

### 3.3 Anti-coagulant and anti-thrombotic effect

Platelet activating factor (PAF), the most potent platelet activator known, has a wide range of biological activities and can be synthesized by a variety of cells including platelets, leukocytes and endothelial cells. It was found that GuHong could significantly increase the contents of tissue-type plasminogen activator (t-PA) and 6-keto prostaglandin F_1α_ (6-keto-PGF_1α_), and decrease the contents of plasminogen activator inhibitor (PAI) and thromboxane B_2_ (TXB_2_) in serum of rats with ischemia reperfusion. The dynamic balance between TXB_2_/6-keto-PGF_1α_ are important factor in regulating vascular wall tension, platelet function, preventing thrombosis and regulating local blood flow. These results showed that GuHong had good anti-thrombotic effects in the treatment of stroke (Shu et al., 2014). Safflower yellow, the main active ingredient in safflower, is extracted from the aqueous extract of safflower and has anti-coagulant pharmacological activity. Safflower yellow could inhibit PAF induced platelet activation and suppress platelet aggregation, release reaction, and increase intracellular free calcium (Zang et al., 2002; Jin et al., 2004; Huang et al., 2012). Experimental data in the literature showed that safflower yellow could significantly prolong plasma prothrombin time (PT), thromboplastin time (TT) and activated partial thromboplastin time (APTT), reduce plasma fibrinogen content and inhibit ADP-induced platelet aggregation in rats. Moreover, it significantly reduced whole blood viscosity, plasma viscosity and erythrocyte aggregation index in rats with blood stasis models (Li et al., 2009). Coagulation factors F7 and F2 were recognized as crucial factors in the extrinsic coagulation pathway. Studies have revealed that GuHong can significantly decrease the mRNA expression of coagulation factors F7 and F2. This suggests that the coagulation cascade regulated by these factors may serve as targets for GuHong’s anti-thrombotic effect (Wang et al., 2022b).

### 3.4 Neuroprotection effect

Neuroprotective therapy is aimed at the main pathological mechanism of ischemic stroke and the biochemical and metabolic disorders of ischemic brain injury through drugs or other means to block cell necrosis, increase cell survival ability, and promote the recovery of neurological function. Neuroprotective agents play a vital role in reducing cell damage following ischemia. Their primary objective are to extend the time window for cerebral perfusion therapy, delay nerve cell death, and alleviate brain dysfunction. (Tuo et al., 2022). Neuroprotection and neurorestoration therapy are the two main drug intervention strategies for ischemic stroke. Neuroprotective therapy can significantly prolong the time window of thrombolytic therapy and reduce cerebral ischemic injury (Zhu et al., 2022). Immunohistochemical staining of rat cerebral tissues revealed a significant increase in the expression of BFGF, VEGF, and TGF-β1 following the administration of GuHong (Zhou et al., 2021). GuHong injection promotes the expression of vascular endothelial growth factor (VEGF-B), nerve growth factor (NGF) and glial cell line-derived neurotrophic factor (GDNF) to reduce nerve damage caused by diabetic peripheral neuropathy (Martel et al., 2017). Studies have revealed that stroke disrupts glycolysis, the TCA cycle, the malate-aspartate cycle, the glutamate-glutamine cycle, nucleic acid metabolism, and phospholipid metabolism in the affected regions of the ischemic hemisphere in rats. However, administration of GuHong injection has been found to regulate the levels of these metabolites, leading to significant improvements in cerebral infarction rate, neurological deficits, cerebral blood flow, and neuronal damage. (Wang et al., 2023).

**FIGURE 4:**
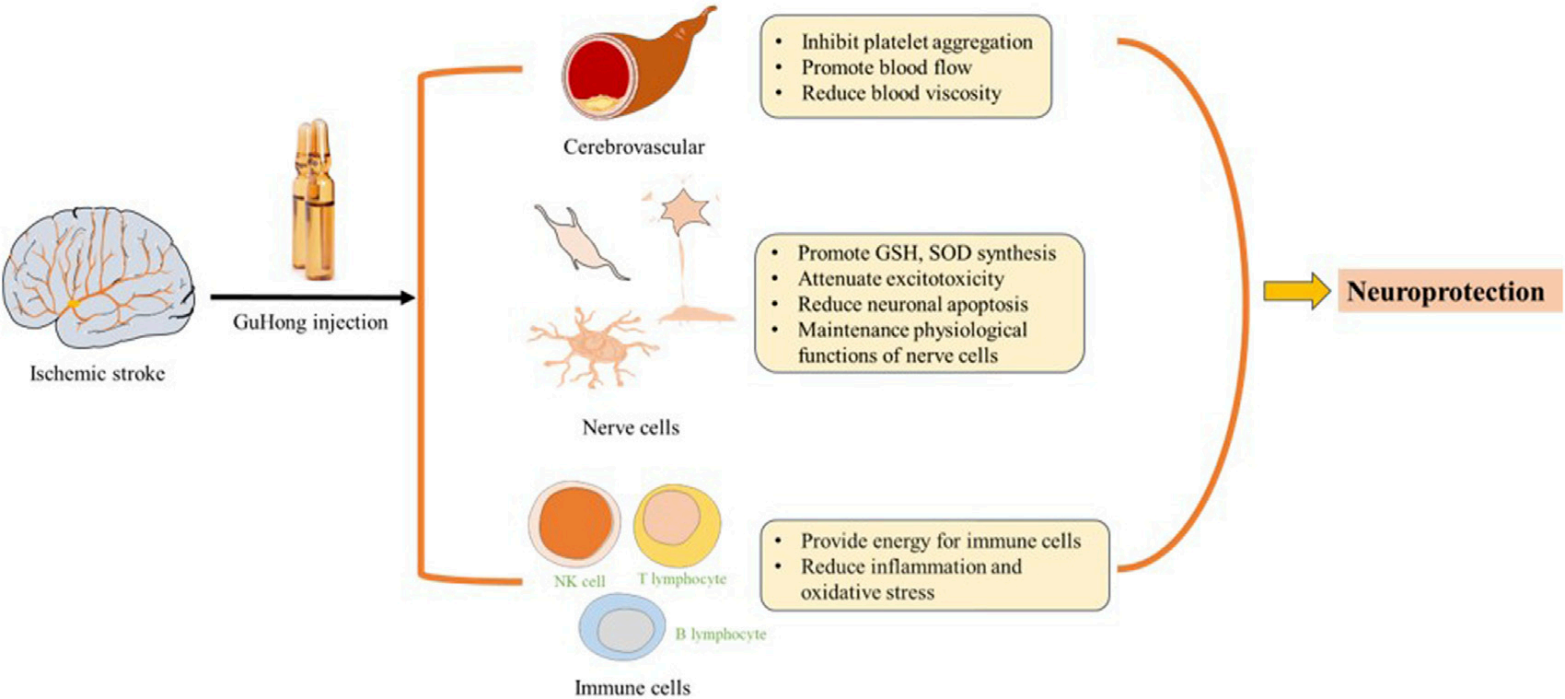
Neuroprotective mechanism of GuHong injection3.5 Anti-apoptosis.

Excitotoxicity is one of the molecular mechanisms of post-ischaemic stroke injury. During cerebral ischemia and hypoxia, the brain experiences an elevation in the release of excitatory neurotransmitters and a disruption in their reuptake due to metabolic abnormalities. This results in the levels of excitatory neurotransmitters escalate rapidly within the ischemic regions of the brain. When the brain is in a state of ischemia and hypoxia, increased excitatory neurotransmitter release and impaired reuptake due to metabolic disorders result in rapidly increasing levels of excitatory neurotransmitters in ischaemic areas of the brain. Ischemic neuronal injury causes a massive release of glutamate, leading to excessive activation of NMDA receptors and a massive influx of Ca^2+^ into cells, resulting in excitotoxic cell death (Orrenius et al., 2003; Maida et al., 2020). According to the literature, Except for NAG, HSYA is the most abundant chemical ingredient in GuHong and is also the quality control ingredient in safflower (Li X. et al., 2017). HSYA protects the hippocampal neurons from excitatory toxic damage by inhibiting NMDARs and regulating the Bcl-2 family as the main component of GuHong (Yang et al., 2010; Wang et al., 2016). HSYA treatment can also significantly attenuate the neurological defects caused by ischemic stroke and reduce the volume of cerebral infarction. To protect the nerves from damage, it has been observed that decreased hippocampal expression levels of LC3, HIF-1, BNIP3, and Notch1 are effective. (Zhang Y. L. et al., 2022). As a neuroprotective agent, NAG has been proved to improve behavioral functions, reduce infarct volume and elevate the number of TH-positive neurons in the substantia nigra (SN) (Zhang et al., 2015). Safflower and NAG in GuHong injection can synergistically play a neuroprotective role, and the specific mechanism is shown in Figure 4.

Apoptosis is a normal physiological phenomenon of genetically controlled cell death to maintain the homeostasis of the internal environment. When cerebral ischemia and reperfusion occur, excessive apoptosis of neurons in brain tissue can largely exacerbate brain injury and cause a series of normal physiological dysfunctions in the body (Radak et al., 2017).

It has been found that a decrease in Bcl-2 or overexpression of Bax causes neuronal apoptosis after cerebral ischemia. (Love, 2003). GuHong injection was effective to upregulate Bax, Caspase-3 and Cleaved-Caspase-3 while increasing Bcl-2 protein expression after continuous administration to acute ischemic stroke rats significantly (Zhou et al., 2021; Wang et al., 2022a). GuHong alleviated brain I/R injury in MCAO rats by decreasing plasma EPO (erythropoietin), HIF-1α (hypoxia-inducible factor-1α), PKC (protein kinase C), upregulating prolyl hydroxylase structural domain 2 (PHD2) protein, downregulating HIF-1α and iNOS protein, and decreasing nicotinamide adenine dinucleotide phosphate oxidase 4 (NOX4) and HIF-1α mRNA, which can regulate apoptosis (Yu et al., 2021).

GuHong injection effectively inhibits the expression of ASK1, JNK, and p38 mRNA. It also decreases the expression of Bax while elevating the expression of Bcl-2, resulting in a reduction in caspase-3 expression and exerting anti-apoptotic effect (Chen et al., 2023). HSYA could inhibit apoptosis in I/R-injured rat penumbra cortical cells by increasing Bcl-2/Bax ratio and this neuroprotective effect may be related to the activation of PI3K/Akt/GSK3β signaling pathway (Yang et al., 2010; Chen et al., 2013). NAG also reduces apoptosis in nigrostriatal neurons by inhibiting the apoptosis-related factor tumor necrosis factor receptor-associated factor 1 (TRAF1) and by upregulating the P-Akt and Bcl-2 signaling pathways, which can increase the number of Th-positive nigrostriatal neurons and reduce neuronal apoptosis (Zhang et al., 2015). Apart from HSYA and NAG, GuHong contains other active substances that contribute to stroke management. These include baicalin, scutellarin, gallic acid, chlorogenic acid, kaempferol, kaempferol-3-O-β-rutinoside, and rutin. These substances work synergistically to target central pathways involved in inflammation and apoptosis, such as NF-κB p65, TNF-α, IL-6, IL-1β, Bax, Bcl-2, and Caspase-3 (Wang et al., 2022a).

## 4 Pharmacokinetics

Pharmacokinetics is the quantitative study of drug absorption, distribution, metabolism and excretion in the body, which is regulated by many factors (such as dose, administration mode, species and drug interactions). Pharmacokinetic studies are also effective way to discover active ingredients and determine quality markers in Chinese medicine. The specific pharmacokinetic parameters of GuHong injection and its components are summarized in Table 3.

**TABLE 3:** Pharmacokinetic parameters of GuHong injection and its components.

Species	Drug/dose	Analyses	Analyte methods	Measure sample	C_max_ (ng/mL)	T_1/2_ (h)	AUC_0-t_ (mg·h/L)	AUC_0-∞_ (mg·h/L)	MRT (h)	Reference
Normal SD rats (n = 6)	GuHong injection, 2.10 mL/kg	HSYA	HPLC	plasma	15.15 ± 0.39	2.29 ± 0.55	47.25 ± 0.45	57.16 ± 3.42	2.10 ± 0.47	Yu et al. (2018)
Normal SD rats (n = 6)	GuHong injection, 2.10 mL/kg	NAG	HPLC	plasma	338.83 ± 7.01	0.78 ± 0.26	1282.41 ± 32.91	1292.41 ± 29.48	1.19 ± 0.04	
MCAO SD rats (n = 6)	GuHong injection, 2.10 mL/kg	HSYA	HPLC	plasma	8.84 ± 0.05	2.83 ± 1.29	31.69 ± 0.36	54.53 ± 13.27	3.64 ± 1.60	
MCAO SD rats (n = 6)	GuHong injection, 2.10 mL/kg	NAG	HPLC	plasma	175.13 ± 86.11	0.57 ± 0.28	435.04 ± 213.19	442.44 ± 216.82	0.92 ± 0.45	
Normal SD rats (n = 6)	Acetyl-L-glutamine 75 mg/kg	NAG	LC-MS/MS	Blood microdialysis	74350 ± 4400	0.74 ± 0.05	358.49 ± 18.49	359.75 ± 18.45	0.93 ± 0.03	Xu et al. (2020)
Normal SD rats (n = 6)	Acetyl-L-glutamine 150 mg/kg	NAG	LC-MS/MS	Blood microdialysis	118610 ± 6670	0.64 ± 0.11	594.74 ± 16.74	595.82 ± 16.61	0.94 ± 0.04	
Normal SD rats (n = 6)	Acetyl-L-glutamine 300 mg/kg	NAG	LC-MS/MS	Blood microdialysis	128250 ± 6240	0.50 ± 0.20	793.88 ± 52.77	797.78 ± 54.73	1.24 ± 0.07	
Normal SD rats (n = 6)	GuHong injection, 10 mL/kg	NAG	LC-MS/MS	Blood microdialysis	118370 ± 6500	0.76 ± 0.22	750.82 ± 64.56	755.12 ± 65.32	1.29 ± 0.06	
Normal SD rats (n = 6)	Acetyl-L-glutamine 75 mg/kg	NAG	LC-MS/MS	Brain microdialysis	7760 ± 500	2.01 ± 0.30	50.14 ± 1.37	58.69 ± 3.48	2.84 ± 0.42	
Normal SD rats (n = 6)	Acetyl-L-glutamine 150 mg/kg	NAG	LC-MS/MS	Brain microdialysis	30570 ± 3330	0.96 ± 0.06	163.15 ± 7.62	165.27 ± 7.32	1.44 ± 0.03	
Normal SD rats (n = 6)	Acetyl-L-glutamine 300 mg/kg	NAG	LC-MS/MS	Brain microdialysis	53440 ± 4710	0.96 ± 0.10	275.07 ± 13.99	278.67 ± 14.04	N.d	
Normal SD rats (n = 6)	GuHong injection, 10 mL/kg	NAG	LC-MS/MS	Brain microdialysis	41480 ± 3340	1.25 ± 0.25	226.34 ± 14.10	241.01 ± 25.23	N.d	
MCAO SD rats (n = 10)	HSYA 4 mg/kg	HSYA	HPLC	plasma	N.d	0.84 ± 0.21	51296.40 ± 7095.60	51645.60 ± 7481.40	1.00 ± 0.10	Chen et al. (2016)
MCAO SD rats (n = 10)	GuHong injection, 10 mL/kg (equal to HSYA 4 mg/kg)	HSYA	HPLC	plasma	N.d	1.06 ± 0.26	95102.40 ± 17421.00	97941.00 ± 20107.80	1.64 ± 0.28	
Normal SD rats (n = 4)	Safflower injection 1 mL/kg	HSYA	LC-MS/MS	plasma	2624.50 ± 660.17	0.47 ± 0.07	2.48 ± 1.31	2.49 ± 1.32	0.78 ± 0.19	Shi et al. (2022)
Normal SD rats (n = 3)	Safflower injection 2 mL/kg	HSYA	LC-MS/MS	plasma	5628.33 ± 405.14	0.64 ± 0.08	5.07 ± 3.29	5.46 ± 2.02	0.97 ± 0.06	
Normal SD rats (n = 3)	Safflower injection 4 mL/kg	HSYA	LC-MS/MS	plasma	14077.33 ± 17.21	0.46 ± 0.12	11.02 ± 1.03	11.45 ± 6.68	0.78 ± 0.11	
Healthy volunteers (n = 12)	HSYA 25 mg/kg	HSYA	LC-MS/MS	plasma	1736 ± 381	4.10 ± 0.42	6.80 ± 1.25	6.89 ± 1.28	N.d	Li et al. (2015)
Healthy volunteers (n = 12)	HSYA 50 mg/kg	HSYA	LC-MS/MS	plasma	3207 ± 582	3.91 ± 0.39	12.66 ± 2.23	6.89 ± 1.28	N.d	
Healthy volunteers (n = 12)	HSYA 75 mg/kg	HSYA	LC-MS/MS	plasma	3603 ± 554	4.18 ± 0.29	16.33 ± 2.13	16.56 ± 2.16	N.d	

Note: HSYA, hydroxysafflor yellow A; NAG, *N*-acetyl-L-glutamine; HPLC, high performance liquid chromatography; LC-MS/MS, liquid chromatography tandem mass spectrometry; C_max_, maximum plasma concentration; T_1/2_, elimination half-life; AUC_0-t_, area under the plasma concentration-time curve from 0 to last measurable time point after dosing; AUC_0-∞_, area under the plasma concentration-time curve from zero to infinity; MRT, mean residence time. N.d., not detected.

According to current literature reports, after the administration of GuHong, most scholars mainly studied the pharmacokinetic characteristics of target compounds, NAG and HSYA in safflower. Yu et al. studied the pharmacokinetics of intravenous glutamine injection (2.1 mg/kg) in healthy and MCAO pathological rats using HPLC analysis method (Yu et al., 2018). The results showed that the main exposed substances in plasma were NAG and HSYA after intravenous glutamine injection. The *C*_max_, *t*_1/2_, AUC_0-∞_ and MRT of HSYA in healthy rats and MCAO model rats were 15.15 ± 0.39 vs*.* 8.84 ± 0.05 μg/L, 137.47 ± 32.91 vs*.* 169.76 ± 77.50 min, 952.89 ± 57.00 vs*.* 909.84 ± 221.11 μg/L*min, 125.81 ± 28.01 vs*.* 218.51 ± 95.87min, respectively. The *C*_max_, *t*_1/2_, AUC_0-∞_ and MRT of NAG in healthy rats and MCAO model rats were 338.83 ± 7.01 vs*.* 175.13 ± 86.11 μg/L, 46.91 ± 15.87 vs*.* 34.48 ± 16.96 min, 21373.54 ± 548.58 vs*.* 7250.72 ± 3553.22 μg/L*min, 71.61 ± 2.59 vs*.* 55.39 ± 27.15 min, respectively. In the rats of MCAO group, the *C*_max_ and AUC_0-∞_ of HSYA and NAG were significantly higher than those of the healthy group. This results indicate that under the pathological condition of MCAO, compounds of GuHong may enter the brain and be utilized due to the disruption of the blood-brain barrier, thereby reducing their exposure in the plasma. Healthy rats were treated with low (75 mg/kg), medium (150 mg/kg) and high (300 mg/kg) doses of NAG and GuHong injection (10 mL/kg, equivalent to 150 mg/kg containing NAG), and the dialysate of blood and brain was collected by LC-MS/MS combined with microdialysis sampling technique. According to the AUC_0-∞, brain_/AUC_0-∞, blood_ results (Liu and Li, 2010), NAG can cross the blood-brain barrier and act on the central nervous system. Comparing its half-life in blood and brain, NAG is eliminated faster in blood than in brain (Xu et al., 2020). Besides, according to the administration of NAG at low, medium and high doses, the dose-exposure relationship of *C*_max_ and AUC_0-∞_ in blood and brain was not proportional (Smith et al., 2000). Healthy volunteers were given 25, 50 and 75 mg/kg HSYA respectively, and the dose-exposure relationship of AUC_0-∞_ was linear (Li et al., 2015). According to the results of the literature, rats were given 4 mg/kg HSYA and 10 mL/kg GuHong injection (equal to HSYA 4 mg/kg), and the *t*_1/2_ and AUC_0-∞_ were 50.11 ± 12.62 vs*.* 63.53 ± 15.84 min, 860.76 ± 124.69 vs*.* 1632.35 ± 335.13 respectively. GuHong injection contains both safflower and NAG, which may have pharmacokinetic matrix effect in the body and affect the pharmacokinetic characteristics of HSYA (Chen et al., 2016).

## 5 Clinical application of GuHong injection

**FIGURE 5:**
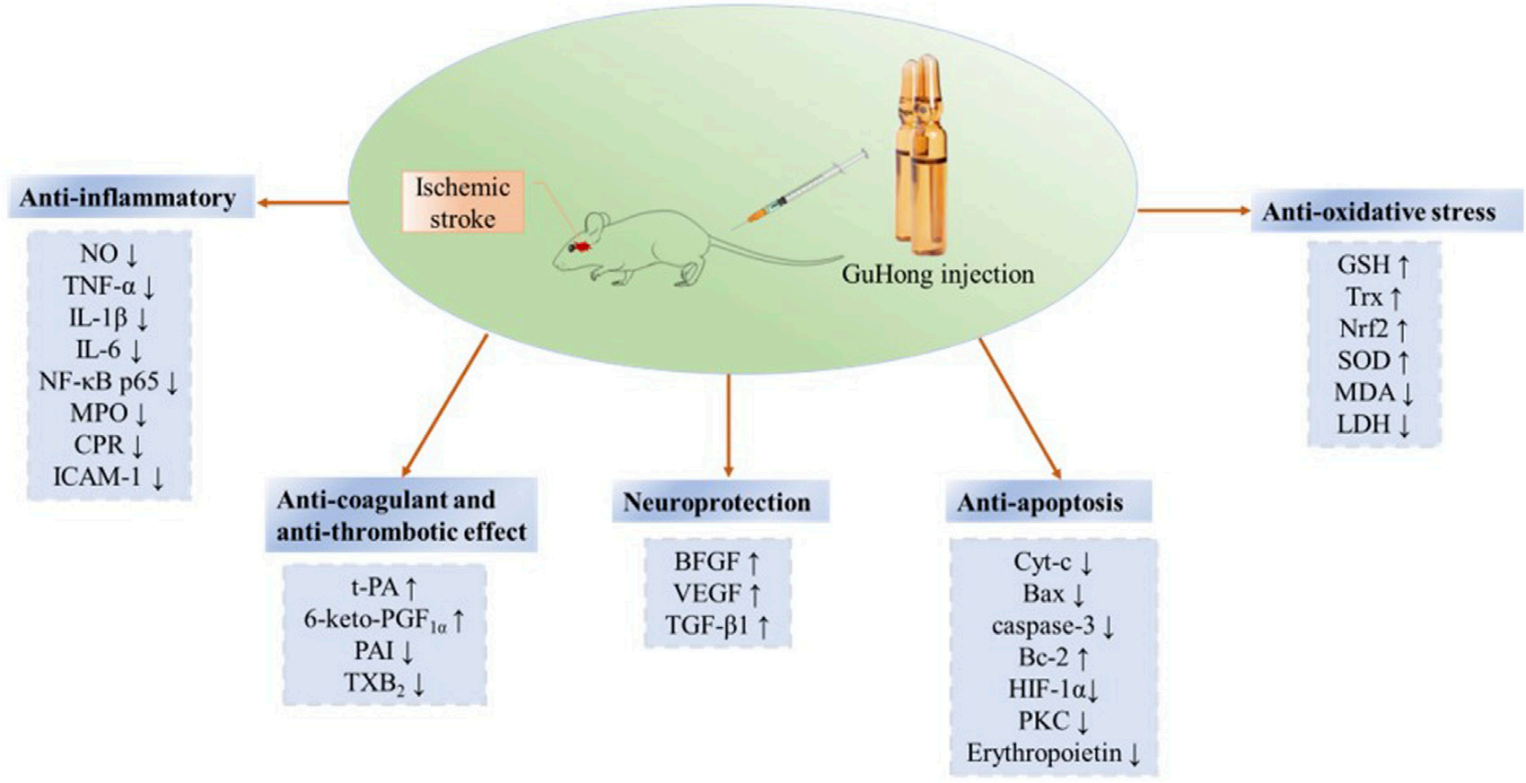
Pharmacological mechanism of GuHong Injection.

GuHong injection was approved for marketing in 2003 and has been in clinical use for about 20 years. The main ingredients are NAG and safflower extract, which complement each other. NAG can improve nerve cell metabolism and brain function; *Carthamus tinctorius L.* is included in the Chinese Pharmacopoeia from the 1963 to 2020 editions and it is a commonly utilized Chinese herb with the effect of promoting blood circulation to remove blood stasis. GuHong has been extensively employed in clinical for the treatment of diverse cardiovascular and cerebrovascular diseases. It exhibits several beneficial pharmacological effects such as enhancing coagulation function, suppressing inflammation, exerting antioxidant properties, preventing neuronal damage, and mitigating ischemia-reperfusion injury. These effects are illustrated in Figure 5. GuHong injection is recommended by the “Expert Consensus on Integrated Chinese and Western Medicine Treatment of Chronic Cerebral Ischemia” (Gao, 2018) and used as intravenous preparation in clinical practice (Liu et al., 2020). According to the results of the meta-analysis of clinical application of GuHong injection, it is mainly used in acute ischemic stroke and vascular cognitive impairment caused by cerebrovascular disease. The summary results of meta-analysis literature on the clinical application of GuHong are shown in Table 4.

**TABLE 4:** The clinical application of GuHong injection.

Disease	Sample size		Gender (M/F)		Age (years)		Medication			Treatment time	Clinical therapeutic effect evaluation	References
	T	C	T	C	T	C	T	C				
Acute ischemic stroke	30	30	16/14	16/14	43–73	43–74	GuHong injection, i.v	MaiLuoNing injection, i.v		14 days	NIH Stroke Scale Glasgow Coma Scale	Cai et al. (2006)
Acute ischemic stroke	42	42	22/20	24/18	47–85	47–83	GuHong injection, i.v	compound danshen injections, i.v		14 days	Clinical neurological deficit score in Chinese stroke patients (1995)	He (2006)
Acute ischemic stroke	50	45	32/18	30/15	60–72	61–75	GuHong injection, i.v	compound danshen injections, i.v		14 days	Scandinavian stroke scale	Wang and Dong (2006)
Acute ischemic stroke	60	60	37/23	35/25	40–72	38–73	GuHong injection, i.v	compound danshen injections, i.v		15 days	Clinical neurological deficit score in Chinese stroke patients (1995)	Li et al. (2007)
Acute ischemic stroke	50	50	24/26	21/29	57–88	61–93	GuHong injection, i.v	Saiviae Miltiorrhizae and Ligustrazine Hydrochloride Injection, i.v		14 days	NIH Stroke Scale	Zhang and Ning (2015)
Acute ischemic stroke	138	143	80/58	93/50	50–73	52–72	Initial therapy + GuHong injection, i.v	Initial therapy		14 days	NIH Stroke Scale, Glasgow Coma Scale Modified Rankin Scale	Zhang (2010)
Acute ischemic stroke	40	40	19/21	20/20	40–75	41–75	Initial therapy + GuHong injection, i.v	Initial therapy		14 days	NIH Stroke Scale, Glasgow Coma Scale Modified Rankin Scale	Jiang et al. (2016)
Acute ischemic stroke	68	68	39/29	41/27	48–85	46–83	Butylphthalide and Sodium Chloride injection + GuHong injection, i.v	Butylphthalide and Sodium Chloride injections, i.v		14 days	NIH Stroke Scale	Li and Zhang (2018)
Acute ischemic stroke	38	38	21/17	23/15	46–82	45–82	Ozagrel Sodium injection + GuHong injection, i.v	Ozagrel Sodium injection, i.v		14 days	NIH Stroke Scale	Sheng (2019)
vascular cognitive impairment	186	143	106/80	83/60	56–60	56–70	GuHong injection, i.v	Dengzhanxixin injection, i.v		14 days	Basic cognitive ability test	Zhao (2006)
vascular cognitive impairment	35	35	18/17	16/19	56–79	60–78	GuHong injection, i.v	Danshen injections, i.v		14 days	Neurological impairment score	Liu (2017)
vascular cognitive impairment	30	30	15/15	18/12	67–85	68–85	Initial therapy + GuHong injection, i.v	Initial therapy		14 days	Montreal Cognitive Assessment	Jia (2019)
vascular cognitive impairment	38	38	24/14	22/16	55–82	54–81	GuHong injection, i.v	Acetamide Pyrrolidone injection, i.v		21 days	Montreal Cognitive Assessment	Wang (2020)
Coronary heart disease	33	33	20/13	21/12	55–81	52–82	Initial therapy + GuHong injection, i.v	Initial therapy		10 days	Resting electrocardiogram	He et al. (2019)
Coronary heart disease	38	38	25/13	27/11	61–76	60–72	Initial therapy + GuHong injection, i.v	Initial therapy		ND	hematological examination	Chen and Xu (2020)
Coronary heart disease	130	130	N.d	N.d	N.d	N.d	GuHong injection, i.v	physiological saline, i.v		10 days	anginal attack frequency Electrocardiogram changes TCM symptoms and signs scores	Zhuang et al. (2020)
Coronary heart disease	58	58	33/25	32/26	51–76	52–75	GuHong injection, i.v	Metoprolol Tartrate Tablets, p.o		15 days	hemorheology	Xu (2020)

Note: T, treatment group; C, control group; M, male; F, female; i. v., intravenous; p. o., peros.

## 6 Discussion and conclusion

In the present review, we systematically summarized the informations about GuHong injection, including the phytochemistry, pharmacokinetics, pharmacological effects and clinical studies. Ischemic stroke is mainly caused by thrombosis, embolism and focal hypoperfusion, which can result in cerebral ischemia and hypoxia. The pathophysiology is complex and can cause a range of responses including energy depletion, excitotoxicity, oxidative stress, blood-brain barrier disruption (BBB), inflammation, necrosis or apoptosis (Campbell et al., 2019). Previous studies have shown that GuHong is a multi-component, multi-target and multi-pathway agent with anti-inflammatory, anti-oxidant, anti-apoptotic and neuroprotective effects. It is mainly used clinically for the treatment of cardiovascular and cerebrovascular diseases. The pharmacokinetics-based identification of these exposure compounds, together with metabolites after dosing GuHong injection, will facilitate uncovering active constituents responsible for the injection’s therapeutic action. GuHong injection has significant advantages in the treatment of ischemic stroke through multi-substance, multi-pathway mechanism of action.

Moreover, although GuHong has shown some efficacy in the treatment of ischemic stroke, there are still many problems and challenges. First of all, there are few literatures available that report the composition spectrum of GuHong, and it is unclear about how many chemical compositions are contained in GuHong injection from safflower. Secondly, there is a lack of overall pharmacodynamic studies on NAG, and it is not possible to state whether it is the original form or the metabolite that exerts the pharmacological effect. Thirdly, Pharmacokinetics of the bioactive components of GuHong are absent in experimental animals, healthy volunteers and patients with ischemic stroke. The available pharmacokinetic studies are insufficient of distribution, metabolism and excretion. Fourthly, multicentre, large-scale, methodologically reliable trials are still needed to verify the efficacy of GuHong in the treatment of ischemic stroke. Finally, more high-quality designed experiments and literature are needed to provide more credible evidence for the effectiveness and safety of GuHong.

In short, it is the first time to systematically summarize the basic information about GuHong, which might provide relatively comprehensive basic data for the related research of GuHong. Although GuHong has shown some efficacy in the treatment of ischemic stroke, there are some scientific gaps that need to be filled currently. More research concerning pharmacokinetics, interactions with other drugs, clinical efficacy and safety, pharmacological mechanisms of bioactive components, and large-scale clinical trials should be conducted in the future.

## Figures and Tables

**Table:** 

**ATP**	Adenosine triphosphate
**BFGF**	Basic fibroblast growth factor
**Bax**	BCL2-associated X protein
**Bcl-2**	B cell leukemia/lymphoma 2
**Cyt-c**	Cytochrome c
**C5AR1**	C5a anaphylatoxin chemotactic receptor 1
**CRP**	C reactive protein
**GSH**	Glutathione
**HIF-1α**	Hypoxia-inducible factor-1α
**H&E**	Hematoxylin and eosin staining
**HSYA**	Hydroxysafflor yellow A
**iNOS**	Inducible NO synthase
**IL-1β**	Interleukin-1β
**ICAM-1**	Intercellular cell adhesion molecule-1
**IL-6**	Interleukin-6
**LDH**	Lactate dehydrogenase
**MPO**	Myeloperoxidase
**MDA**	Malondialdehyde
**MMP-9**	Matrix metalloproteinase-9
**NF-κB p65**	Nuclear factor-κB p65
**Nrf2**	Nuclear factor (erythroid-derived 2)-like 2
**NO**	Nitric oxide
**NAG**	N-acetyl-L-glutamine
**PAI**	Plasminogen activator inhibitor
**PKC**	Protein kinase C
**SOD**	Superoxide dismutase
**TNF-α**	Tumor necrosis factor-α
**Trx**	Thioredoxin
**TXB2**	Thromboxane B2
**TGF-β1**	Transforming growth factor-β1
**t-PA**	Tissue-type plasminogen activator
**VEGF**	Vascular endothelial growth factor
**TCA**	Tricarboxylic Acid Cycle
**VCAM-1**	Vascular cell adhesion protein 1
**6-Keto-PGF1α**	6-keto prostaglandin F1α
**8-OHdG**	8-hydroxy-2′-deoxyguanosine
